# Targeting MyD88 Downregulates Inflammatory Mediators and Pathogenic Processes in PBMC From DMARDs-Naïve Rheumatoid Arthritis Patients

**DOI:** 10.3389/fphar.2021.800220

**Published:** 2021-12-23

**Authors:** Sergio Ramirez-Perez, Edith Oregon-Romero, Itzel Viridiana Reyes-Perez, Pallavi Bhattaram

**Affiliations:** ^1^ Department of Orthopaedics, Emory University School of Medicine, Atlanta, GA, United States; ^2^ Department of Cell Biology, Emory University School of Medicine, Atlanta, GA, United States; ^3^ Biomedical Sciences Research Institute (IICB), University of Guadalajara, Guadalajara, Mexico; ^4^ Department of Molecular Biology and Genomics, University of Guadalajara, Guadalajara, Mexico

**Keywords:** rheumatoid arthritis, MyD88, DMARDs, downregulation, inflammatory mediators, pathogenic processes

## Abstract

MyD88-dependent intracellular signalling cascades and subsequently NF-kappaB-mediated transcription lead to the dynamic inflammatory processes underlying the pathogenesis of rheumatoid arthritis (RA) and related autoimmune diseases. This study aimed to identify the effect of the MyD88 dimerization inhibitor, ST2825, as a modulator of pathogenic gene expression signatures and systemic inflammation in disease-modifying antirheumatic drugs (DMARDs)-naïve RA patients. We analyzed bulk RNA-seq from peripheral blood mononuclear cells (PBMC) in DMARDs-naïve RA patients after stimulation with LPS and IL-1β. The transcriptional profiles of ST2825-treated PBMC were analyzed to identify its therapeutic potential. Ingenuity Pathway Analysis was implemented to identify downregulated pathogenic processes. Our analysis revealed 631 differentially expressed genes between DMARDs-naïve RA patients before and after ST2825 treatment. ST2825-treated RA PBMC exhibited a gene expression signature similar to that of healthy controls PBMC by downregulating the expression of proinflammatory cytokines, chemokines and matrix metalloproteases. In addition, B cell receptor, IL-17 and IL-15 signalling were critically downregulated pathways by ST2825. Furthermore, we identified eight genes (*MMP9*, *CXCL9*, *MZB1*, *FUT7*, *TGM2*, *IGLV1-51*, *LINC01010*, and *CDK1*) involved in pathogenic processes that ST2825 can potentially inhibit in distinct cell types within the RA synovium. Overall, our findings indicate that targeting MyD88 effectively downregulates systemic inflammatory mediators and modulates the pathogenic processes in PBMC from DMARDs-naïve RA patients. ST2825 could also potentially inhibit upregulated genes in the RA synovium, preventing synovitis and joint degeneration.

## Introduction

Rheumatoid arthritis (RA) is an autoimmune, inflammatory-chronic and systemic disease of multifactorial etiology. The presence of circulating autoantibodies and increased production of inflammatory mediators are the most important immunological changes in RA. Inflammatory mediators play an essential role in the joint pathology of RA by promoting synovitis, articular cartilage degeneration and bone loss. These critical mediators include the tumor necrosis factor (TNF)-α, interleukin (IL)-1β, and IL-6 (Aletaha and Smolen, 2018; Smolen et al., 2018). In order to mitigate the joint damage caused by systemic inflammation in RA patients, chemical and biological therapies directed against these crucial cytokines have been established. The development of biological disease-modifying antirheumatic drugs (DMARDs), including anti-TNF, anti-IL-1 and anti-IL-6 antibodies, have contributed to ameliorating patients’ disease status and delaying the RA progression (Brzustewicz and Bryl, 2015). However, a significant proportion of RA patients fail to show the desired response to the biological DMARDs, highlighting the need to develop additional therapeutics (Aletaha, 2020).

The proinflammatory cytokine IL-1β, is among the critical mediators of inflammatory damage in RA. It exerts its activity via interaction with its receptors (IL-1R), which subsequently leads to the presentation of an intracellular Toll-IL-1-receptor (TIR) domain and activation of downstream signalling cascades that converge into large scale transcriptomic changes (Migliorini et al., 2020; Haque et al., 2021). Myeloid Differentiation Primary Response 88 (Myd88) is a significant constituent of the signalling cascade downstream of IL-1β-IL-1R interaction, which ultimately converges with the canonical NF-κB signalling pathway leading to the further amplification of the inflammatory mediator production in RA (Avbelj et al., 2011; Chen et al., 2020). Another important proinflammatory promoter is the bacterial lipopolysaccharide (LPS), which activates MyD88-dependent intracellular signalling cascades and, subsequently NF-κB-mediated transcription, leading to the activation of inflammatory processes underlying RA (Balka and De Nardo, 2019).

Targeting MyD88 by the effect of the synthetic chemical compound ST2825 has shown a significant decrease in the production of proinflammatory cytokines such as IL-1β, IL-6, TNF-α and IL-12 after LPS stimulation in macrophages (Long et al., 2018), kidney epithelial cells (Qi et al., 2016) and PBMC from healthy subjects (Ramírez-Pérez et al., 2020). The precise ST2825 mechanism-of-action for inhibiting MyD88 is mediated by interfering with homo-oligomerization of BB loop in the MyD88 TIR domain and thereby affecting its dimerization and downstream signalling activation (Loiarro et al., 2007; Loiarro et al., 2013; Chen et al., 2020). In addition, different studies have reported that ST2825 leads to a decrease in the recruitment and activation of IRAK1, IRAK4, TRAF6, IKK complex, p-BTK, p-IκB, NF-κB (p65) and HIF-1α, factors involved in the activation of inflammatory processes (Qi et al., 2016; Yao et al., 2016; Yang et al., 2018; Wang et al., 2019). Together, these studies suggest that inhibition of MyD88 is a potential RA therapeutic strategy. We, therefore, initiated this study to obtain an indepth understanding of the effect of MyD88 inhibition by ST2825 on systemic inflammation generated by the peripheral blood mononuclear cells (PBMC) from RA patients. In order to decipher the effect of MyD88 inhibition independent of DMARDs, we performed our studies on DMARDs-naïve RA patients. Dysregulated pathways and the expression of genes involved in inflammation were evaluated by performing a transcriptomic analysis of healthy and RA PBMC sensitized to various proinflammatory stimuli. We thus identified the MyD88-dependent immunopathological gene expression signature that is downregulated by its specific inhibitor ST2825. Our studies on DMARDs-naïve patients might provide invaluable information on transcriptional changes that contribute to identifying specific immunopathological pathways implicated in the presentation and progression of RA.

## Materials and Methods

### Study Design

We aimed to identify inflammatory mediators and fundamental signalling pathways in RA, which cause sustained inflammation after stimulation with important mitogens and proinflammatory cytokines such as LPS and IL-1β. The potential effect of ST2825 as an inhibitor of MyD88-dependent inflammatory mechanisms could represent a critical strategy to modulate the inflammatory process in RA. Our working hypothesis is that the chemical molecule ST2825 inhibits the signalling pathways mediated by the activation of peripheral blood mononuclear cells (PBMC) stimulated with bacterial lipopolysaccharides (LPS) and recombinant human IL-1β (hrIL-1β) in treatment-naïve RA patients.

### Reagents

LPS from *Escherichia coli* (CAT-L-2880, SIGMA®), Recombinant Human IL-1 beta/IL-1F2 Protein (201-LB-005, R&D Systems®), and ST2825 Inhibitor of MyD88 dimerization (Cat. No. A3840, APExBIO) were used for PBMC stimulation. All reagents were reconstituted according to the manufacturer’s instructions.

### PBMC and Cell Culture

PBMC samples from DMARDs-naïve RA patients and healthy subjects were purchased from STEMCELL Technologies Inc. and Precision For Medicine Inc. (Supplementary Table S1). Frozen PBMC were thaw according to STEMCELL Technologies instructions. PBMC were cultured in 6-well plates after the density adjustment at 1×10^6^ cells/mL (final volume of 2000 μL). A serum-free system was implemented for PBMC culturing by using X-VIVO™ 15 Hematopoietic Serum-Free Culture Media (Lonza) supplemented with 1% penicillin/streptomycin. PBMC were cultured for 48 h before stimulation. Subsequently, PBMC stimulation was performed by adding LPS (30 ng/ml), LPS (30 ng/ml) plus ST2825 (30 μM), rhIL-1β (10 ng/ml), rhIL-1β (10 ng/ml) plus ST2825 (30 μM) or ST2825 (30 μM). Unstimulated PBMC were taken as the control group. The MyD88 inhibitor ST2825 was added to the corresponding well 30 min before stimulation with LPS or rhIL-1β. A treatment concentration of 30 μM ST2825 was chosen based on our previous study in healthy PBMC (Ramírez-Pérez et al., 2020). Experiments were done in duplicates, and PBMC were incubated for 24 h at 37°C in a humidified 5% CO_2_ atmosphere.

### Bulk RNA Sequencing

PBMC were collected 24 h after stimulation, and total RNA was extracted and purified using Direct-zol RNA MicroPrep (Zymo Research) following manufacturers’ protocol. RNA quality and quantity were assessed using a 2100 Bioanalyzer (Agilent Technologies). Only samples with an RNA integrity number (RIN) > 7 were used. Libraries were generated from 250 ng RNA using TruSeq Stranded Total RNA Sample Prep Kit (Illumina). Sequencing was carried out using the NovaSeq 6000 system (Novogene UC Davis Sequencing Center, Novogene Corporation Inc.). FASTQ files from these samples were uploaded in Strand NGS software (version 4.0) for analysis (Supplementary Table S3). Paired-end reads were mapped to the hg19 human genome assembly. RNA levels were normalized using DESeq. The lower cut-off for RNA levels was = 2 NRPKM (normalized reads per kilobase of exon model per million mapped sequence).

### Bioinformatics Analysis of RNA Sequencing Data

Differentially expressed (DE) genes were identified through Strand NGS software (version 4.0). QIAGEN Ingenuity Pathway Analysis (IPA) was used to identify canonical pathways, upstream regulators, predicted diseases and functions dysregulated between different conditions in our study. Data visualization and DE analysis was performed in GraphPad Prism version 9. Networks of relevant DE genes were identified and analyzed in STRING version 11.5 and Cytoscape version 3.8.2. For bulk RNA-seq data analysis from the Accelerating Medicines Partnership (AMP) (Zhang et al., 2019), synovial tissue was obtained from 14 OA, 17 leukocyte-poor RA and 18 leukocyte-rich RA, synovial cell populations were sorted by flow cytometry. RNA-seq from AMP was performed on sorted cells using the following surface markers: CD45^−^ Podoplanin^+^ for fibroblasts, CD45^+^ CD14^+^ for monocytes, CD45^+^ CD3^+^ for T cells, and CD45^+^ CD3^−^ CD19^+^ for B cells; data were visualized using Immunogenomics.io.

### Statistical Analysis

Differential gene expression changes in RNA-seq were calculated by Audic Claverie test and Benjamini-Hochberg false discovery rate (FDR) for multiple testing corrections. Hierarchical cluster analysis of biological replicates was performed on normalized intensity values from RNA sequenced samples; similarities were determine by using Euclidean distance measure and Wards methods. For bulk RNA-seq data analysis from the Accelerating Medicines Partnership (AMP) Kruskal–Wallis test was performed to observe differences among groups. The p-value cut-off was set at 0.05.

## Results

### Identification of Gene Expression Signatures and Distinct Canonical Pathways in DMARDs-Naïve RA Patients

**FIGURE 1:**
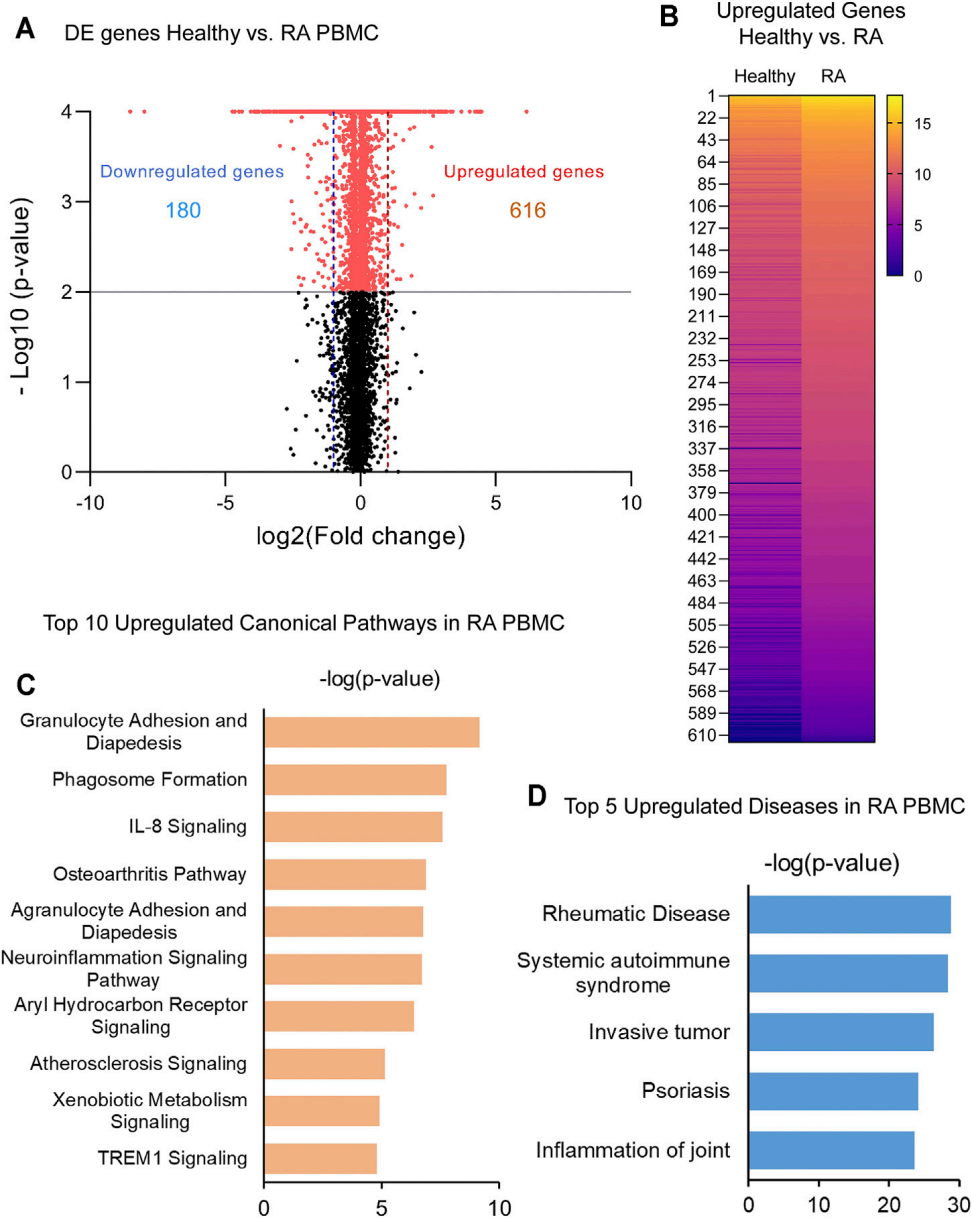
Identification of upregulated genes and canonical pathways in DMARDs-naïve RA patients. **(A)** Volcano plot shows differentially expressed genes between unstimulated PBMC from healthy subjects vs. DMARDs-naïve RA patients (RA). Downregulated genes are shown on the left hand (doted blue line represents 2-fold change) and upregulated genes are shown on the right hand (doted red line represents 2-fold change) of the volcano plot. Grey line represents cut-off of p-value = 0.05. **(B)** Upregulated genes from healthy subjects vs. RA were included in the heatmap (averaged expression patterns of biological replicates are shown). **(C)** Top 10 canonical pathways and **(D)** top 5 of IPA-predicted disease associations were identified from upregulated genes between healthy subjects and DMARDs-naïve RA PBMC.

In order to identify critical gene expression signatures in PBMC from DMARDs-naïve RA patients, differential expression analysis was performed (Figure 1A, Supplementary Figure S1A). We identified 796 differentially expressed (DE) genes by 2-fold change (*p* < 0.05) between RA patients and healthy subjects. The analysis showed 180 downregulated and 616 upregulated genes. Upregulated genes are exemplified in the heatmap (Figure 1B) and the top 5 pathways associated with the upregulated genes identified by IPA analysis are shown in Figure 1C. Interestingly, granulocyte adhesion, phagosome formation, IL-8 signalling, osteoarthritis pathway and neuroinflammation were important pathways associated with DMARDs-naïve RA patients. Furthermore, DE genes were used to identify IPA-predicted disease associations and functions upregulated in DMARDs-naïve RA patients compared with healthy subjects (Figure 1D) and as expected the top 5 IPA-predicted disease associations related to the upregulated genes were directly associated with RA or rheumatic disease processes, which fortify the successful characterization of samples used for this study.

### MyD88 Inhibition Significantly Modulates Gene Expression and Pathogenic Features in PBMC From DMARDs-Naïve RA Patients

**FIGURE 2:**
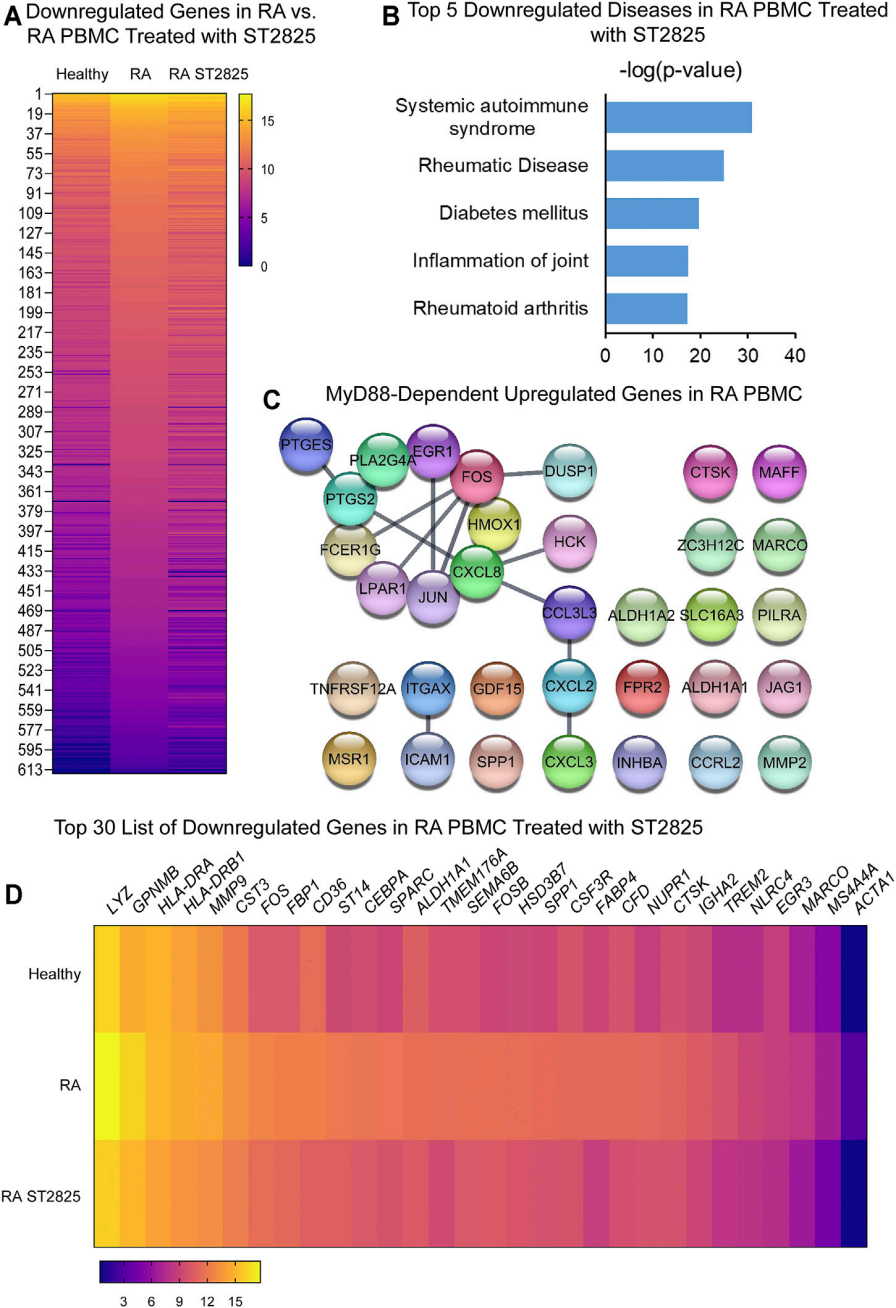
MyD88 inhibition by ST2825 downregulates critical molecules and IPA-predicted disease associations in PBMC from DMARDs-naïve RA patients. **(A)** Transcriptomic signatures from healthy subjects, RA and RA PBMC treated with ST2825 are shown in the heatmap (averaged expression patterns of biological replicates are shown). **(B)** MyD88-predicted target gene network was identified from upregulated genes expressed in RA PBMC vs. healthy subjects. **(C)** Top 5 of IPA-predicted disease associations were determined based on downregulated genes by effect of ST2825. **(D)** Top 30 downregulated genes by effect of ST2825 involved in the inflammatory response are illustrated in the heatmap (averaged expression patterns of biological replicates are shown).

To address specific MyD88 dimerization inhibition, we took advantage of the synthetic chemical compound ST2825, which has previously been reported in several cell studies to downregulate important MyD88-dependent inflammatory signalling pathways. PBMC from DMARDs-naïve RA patients were treated with ST2825, and DE genes were identified. Hierarchical cluster analysis of normalized gene expression from DMARDs-naïve RA after ST2825 treatment is shown in Supplementary Figures S3A–B. Our analysis revealed 631 DE genes between DMARDs-naïve RA patients before and after ST2825 treatment (Figure 2A). The most interesting finding we observed from this analysis was that ST2825-treated RA PBMC exerted a gene expression signature similar to that observed in healthy controls by downregulating the expression of several genes such as *C1QA* (Complement C1q subcomponent subunit A), *C2* (Complement C2), *CR1* (Complement receptor type 1), *CCL22* (C-C motif chemokine 22), *CX3CR1* (CX3C chemokine receptor 1), and *CXCL9* (C-X-C motif chemokine 9). However, ST2825 treatment did not completely normalize RA expression values to the levels seen in healthy control PBMC, where several upregulated genes were downregulated by the effect of MyD88 inhibition. Another exciting finding was attributed to the top 5 downregulated IPA-predicted disease associations in PBMC from DMARDs-naïve RA patients by the effect of ST2825 (Figure 2B), where systemic autoimmune syndrome, rheumatic disease, joint inflammation, and rheumatoid arthritis were significantly inhibited. These results strongly suggest that ST2825 successfully modulates crucial pathogenic hallmarks of RA. We next performed an *in silico* upstream regulatory analysis to identify the main upstream regulators of those upregulated genes in DMARDs-naïve RA patients *vs.* healthy controls. Our analysis found that 34 upregulated genes from DMARDs-naïve RA patients were predicted to be activated by MyD88 (z-score = 5.384; *p*-value = 1.23E-16), which we referred to as MyD88-dependent genes (Figure 2C) (Interaction confidence = 0.9; PPI enrichment *p*-value < 1.1E-13). To determine the effectiveness of ST2825 in reverting the inflammatory transcriptome of the RA PBMC, we compared the top 30 genes that were downregulated by ST2825 in RA PBMC (Figure 2D). The analysis indeed revealed that ST2825 reverted the expression of different transcription factor subunits, receptors, and enzymes involved in inflammatory pathways to levels similar in healthy PBMC. These included *MMP9* (Matrix metalloproteinase-9), *FOS* (Fos proto-oncogene), *ALDH1A1* (Aldehyde Dehydrogenase 1 Family Member A1), *SPP1* (Secreted Phosphoprotein 1), *CTSK* (Cathepsin K), *NLRC4* (NLR family CARD domain-containing protein 4), and *MARCO* (Macrophage Receptor With Collagenous Structure). Downregulated genes by effect of ST2825 are mainly expressed by classical, non-classical and intermediate monocytes, as well as myeloid and plasmacytoid dendritic cells (DCs). In addition, other PBMCs such as natural killer (NK), T and B cells are able to express these genes as well. Principal component analysis (PCA) of the gene expression data from DMARDs-naïve RA patients shows that ST2825 treatment clustered similarly in principal component space. Therefore, these data demonstrate a similar molecular signature of gene expression after MyD88 inhibition (Supplementary Figure S1A). Canonical pathway analysis of genes downregulated by ST2825 identified IL-15 signalling, B cell receptor signalling, communication between innate and adaptive immune cells, dendritic cell maturation, and complement system activation as the targets of ST2825 (Supplementary Figure S1B). Together, these data show that inhibition of MyD88 has the potential to mitigate and, to some extent, revert multiple critical pathogenic features that drive RA pathogenesis.

### Targeting MyD88 Effectively Downregulates Inflammatory Gene Expression Signatures and Pathogenic Processes in LPS-Stimulated PBMC From DMARDs-Naïve RA Patients

**FIGURE 3:**
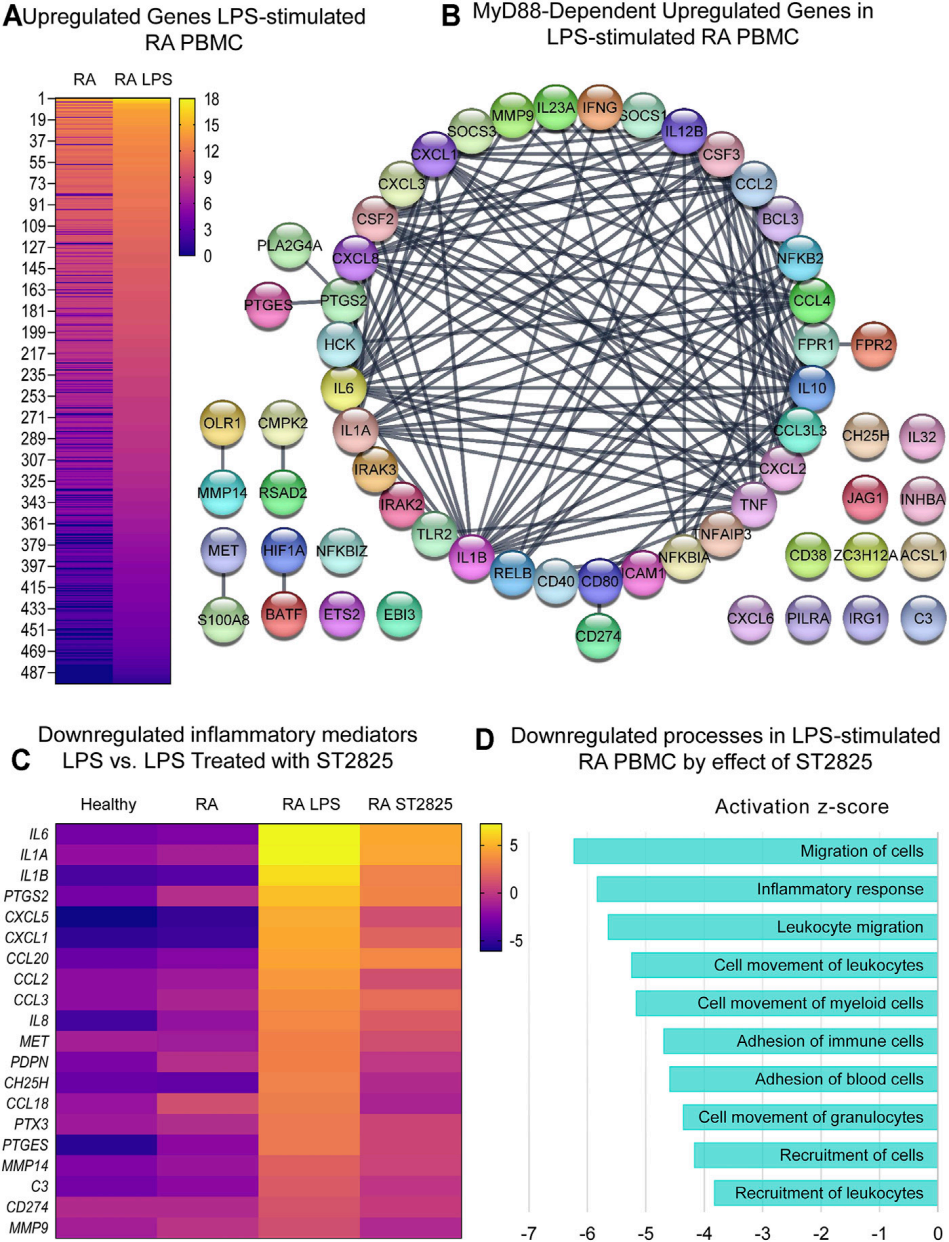
MyD88 inhibition by ST2825 downregulates key inflammatory mediators and pathogenic processes in LPS-stimulated PBMC from DMARDs-naïve RA patients. **(A)** Transcriptomic signatures from RA PBMC and RA LPS-stimulated PBMC are presented in the heatmap (averaged expression patterns of biological replicates are shown). **(B)** MyD88-predicted target gene network was identified from upregulated genes expressed in RA PBMC vs. RA LPS-stimulated PBMC. **(C)** Top 20 downregulated inflammatory mediators by effect of ST2825 are illustrated in the heatmap (averaged expression patterns of biological replicates are shown). **(D)** Top 10 downregulated pathogenic processes were determined based on downregulated genes by effect of ST2825.

Since RA PBMCs are constantly under the influence of proinflammatory mediators, we wanted to determine if MyD88 inhibition has the potential to inhibit the deleterious effects of LPS stimulation. Hierarchical cluster analysis of normalized expression from DMARDs-naïve RA LPS-stimulated PBMC treated with ST2825 is shown in Supplementary Figures S3C,D. As expected, LPS induced 950 DE genes; 454 downregulated genes and 496 upregulated genes were identified on LPS-stimulated PBMC compared with unstimulated cells from DMARDs-naïve RA patients. Upregulated genes are presented in Figure 3A. A subsequent analysis established the top 10 upregulated canonical pathways by the effect of LPS; granulocyte adhesion and diapedesis, IL-17 signalling, TREM1 signalling, and tumor microenvironment were observed in our study (Supplementary Figure S2A). Upstream regulatory analysis indeed predicted that 60 of the LPS upregulated genes were downstream targets of MyD88. The protein interaction network predicted for these 60 MyD88-dependent genes is illustrated in Figure 3B at an Interaction confidence = 0.900; PPI enrichment *p*-value < 1.0E-16. We next evaluated the effect of ST2825 on LPS-stimulated PBMC. We identified 471 DE genes between LPS-stimulated RA PBMC and LPS-stimulated RA PBMC treated with ST2825. The analysis also showed 121 up and 350 downregulated genes. The most relevant finding was the downregulation of crucial inflammatory mediators such as proinflammatory cytokines, chemokines, and matrix metalloproteinases (MMP) by the effect of ST2825 (Figure 3C). Of importance, several MyD88-dependent genes such as the cytokines *IL6*, *IL1A*, *IL1B*, *PTGS2*, chemokines *CXCL1*, *CCL2*, matrix-degrading enzymes *MMP14*, *MMP9*, and complement *C3* were downregulated by ST2825. The significantly downregulated processes (-3 to -6 z-scores) were associated with (Figure 3D) migration of cells, inflammatory response, leucocyte migration, adhesion of immune cells, and recruitment of leukocytes. Canonical pathway analysis further supported these findings by revealing that B cell receptor, IL-15 and IL-17 signalling were critically downregulated pathways by the effect of ST2825 (Supplementary Figure S2B). Together, these findings suggest that ST2825 effectively downregulates MyD88-dependent gene expression and pathogenic processes orchestrated by the effect of LPS in PBMC from DMARDs-naïve RA patients.

### ST2825 Downregulates IL-1β-Dependent Inflammatory Response in PBMC From DMARDs-Naïve RA Patients

**FIGURE 4:**
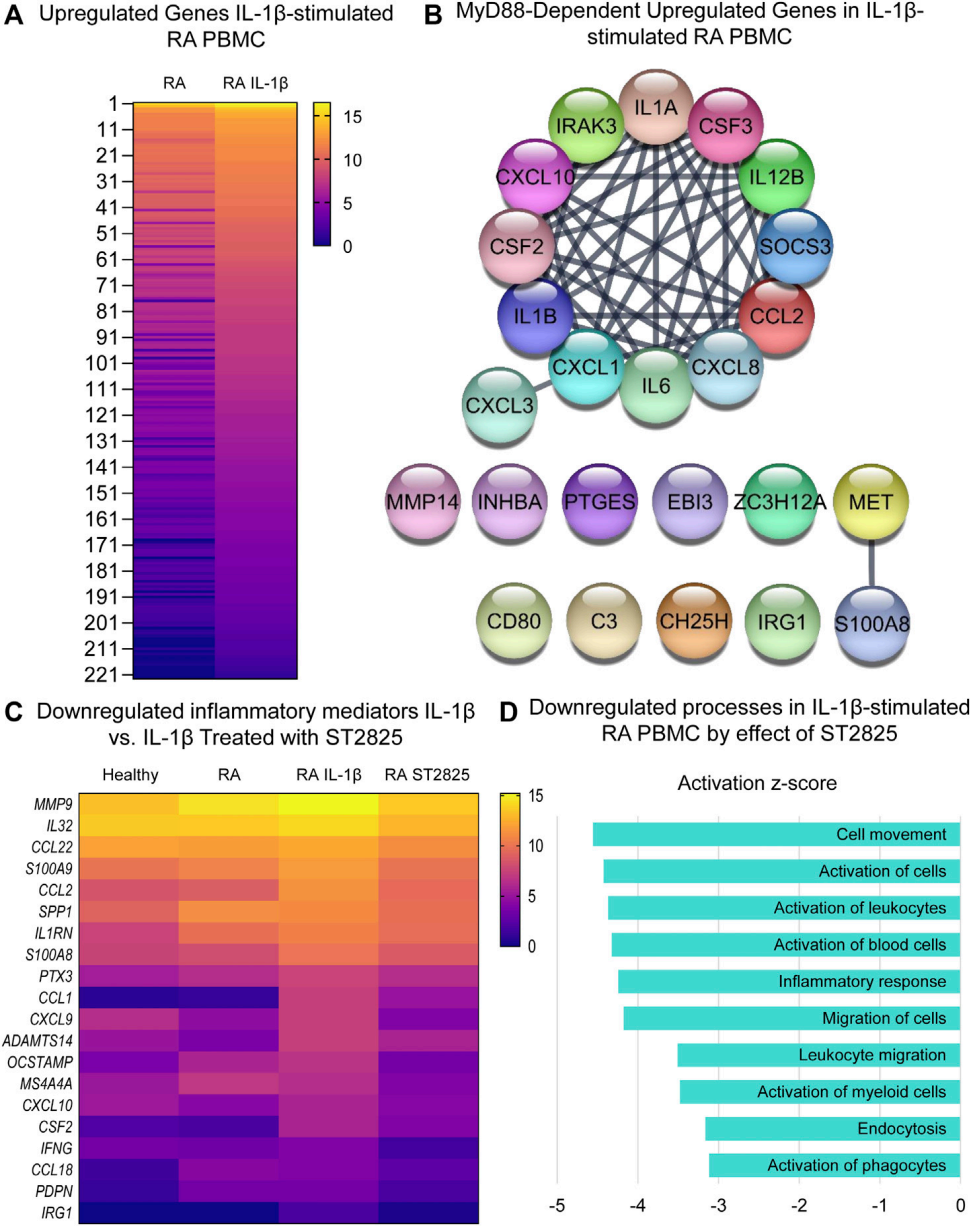
MyD88 inhibition by ST2825 downregulates key inflammatory mediators and pathogenic processes in IL-1β-stimulated PBMC from DMARDs-naïve RA patients. **(A)** Transcriptomic signatures from RA PBMC and RA IL-1β-stimulated PBMC are presented in the heatmap (averaged expression patterns of biological replicates are shown). **(B)** MyD88-predicted target gene network was identified from upregulated genes expressed in RA PBMC vs. RA IL-1β-stimulated PBMC. **(C)** Top 20 downregulated inflammatory mediators by effect of ST2825 are exemplified in the heatmap (averaged expression patterns of biological replicates are shown). **(D)** Top 10 downregulated pathogenic processes were determined based on downregulated genes by effect of ST2825.

Since IL-1β is an important cytokine capable of activating pathogenic mechanisms upstream of MyD88 in RA, we asked whether ST2825 effectively inhibits the pathological gene expression induced by IL-1β on DMARDs-naïve RA PBMC. Hierarchical cluster analysis of normalized expression from DMARDs-naïve RA IL-1β-stimulated PBMC treated with ST2825 is shown in Supplementary Figures S3E,F. IL-1β-stimulation resulted in 375 DE genes. Heatmap illustrates gene expression signatures from 222 upregulated genes by the effect of IL-1β **(** Figure 4A **)**. The main upregulated canonical pathways associated with the upregulated gene signature were granulocyte adhesion and diapedesis, IL-17 signalling in psoriasis and RA, and pattern recognition receptor signalling (Supplementary Figure S2C). Besides, analysis of upstream regulators predicted twenty-four IL-1β target genes to be MyD88-dependent (z-score = 4.786, *p*-value = 9.33E-20), and were predicted to form an interactive protein network at a high degree of statistical confidence **(** Figure 4B **,** confidence = 0.900 and a PPI enrichment *p*-value < 1.0E-16). Similar to the LPS treatment, heatmap analysis revealed that ST2825 was at least partially successful in reverting the IL-1β-induced transcriptomic changes to be comparable to healthy PBMC (Figure 4C). The global inhibitory effect of ST2825 was evident from the downregulated processes (Figure 4D), which included: cell movement, activation of cells, inflammatory response, leucocyte migration, endocytosis, and activation of phagocytes. In addition, IL-15 signalling, B cell receptor signalling, granulocyte adhesion and diapedesis, and the role of macrophages, fibroblasts and endothelial cells in RA were the main downregulated canonical pathways identified in this study (Supplementary Figure S2D). Overall, our observations suggest that dimerization inhibition of MyD88 is an effective target for downregulating the IL-1β-dependent inflammatory response in PBMC from DMARDs-naïve RA patients.

### Targeting MyD88 Could Potentially Inhibit Upregulated Genes in the RA Synovium

**FIGURE 5:**
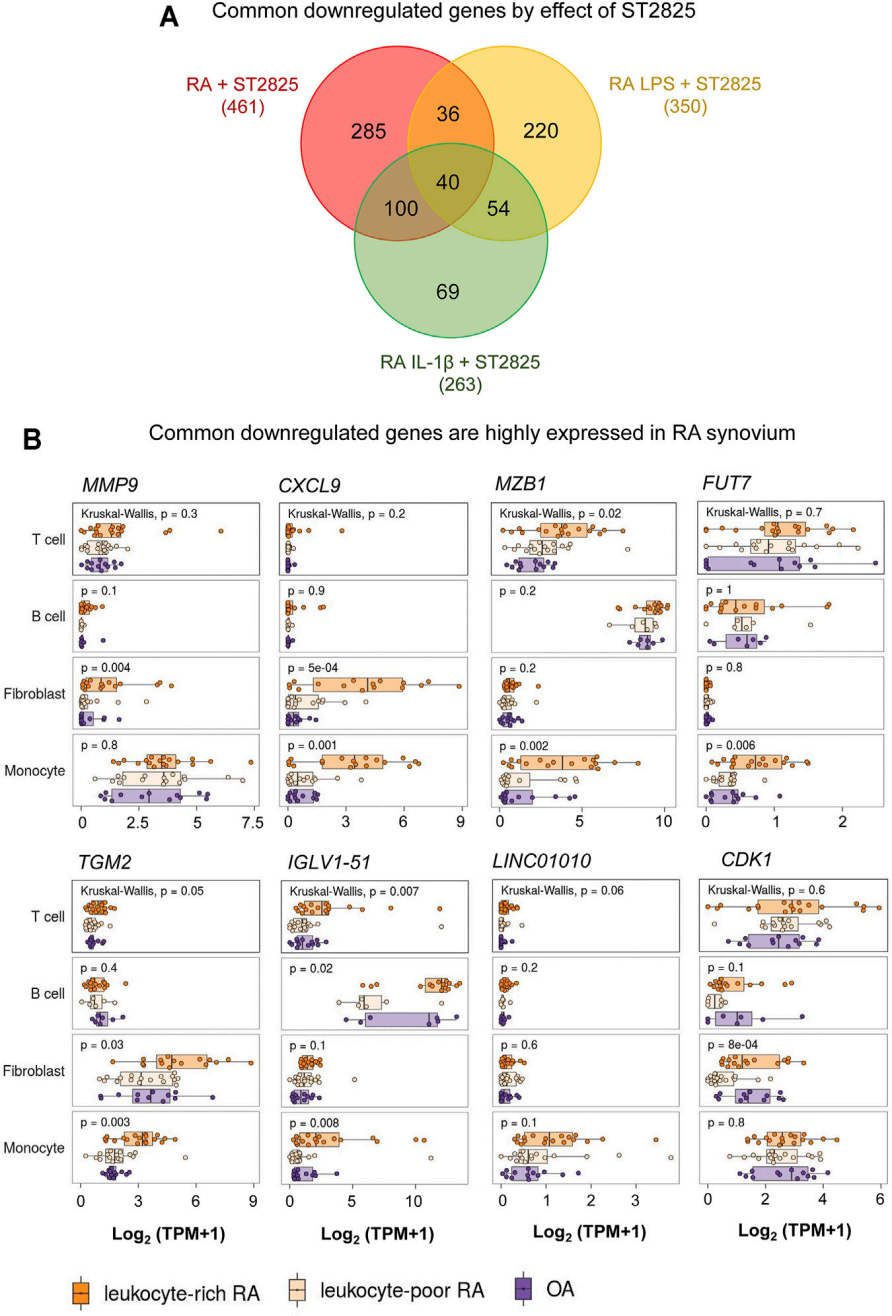
RNA-seq comparison reveals that highly expressed genes in RA synovium could be potentially downregulated by ST2825. **(A)** Venn diagram shows overlap among all sets of downregulated genes from RA PBMC (red), RA LPS-stimulated PBMC (yellow), and RA IL-1β-stimulated PBMC (green) after treatment with ST2825. **(B)** Eight common downregulated genes by effect of ST2825 identified from RA PBMC were highly expressed by different cell types in the synovium of leukocyte-rich RA (orange), leukocyte-poor RA (tan) and OA (purple) patients.

The *in vivo* inflammatory environment constitutes multiple types of inflammatory mediators that feed forward on to the MyD88 and NF-κB pathways and their targets. We, therefore, hypothesized that the most clinically relevant targets of ST2825 could overlap between the LPS and IL-1β. By overlapping the genes downregulated by ST2825 among different treatment conditions tested in this study we identified 40 common genes **(** Figure 5A, Supplementary Table S2). Since, joint degeneration and synovitis are a major part of RA pathology, we asked whether the 40 common ST2825 target genes could also play a role in the RA synovium. Taking advantage of a published RNA-seq data set from synovial tissue cells freshly sorted from RA and OA patients (Zhang et al., 2019), we found that eight of the 40 common ST2825 target genes were highly expressed in the inflammatory leukocyte-rich RA compared to the OA synovium (Figure 5B). It is relevant to highlight that some genes such as *MMP9* and *CXCL9* essential for maintaining and promoting inflammatory and pathogenic processes in RA were upregulated in fibroblasts and monocytes from the inflammatory leukocyte-rich RA synovium. We also identified six genes that are highly expressed in the various cell types of the inflamed synovium for which a specific role has not yet been attributed in the context of RA; *MZB1* (Marginal zone B and B1 cell-specific protein), *FUT7* (Fucosyltransferase 7), *TGM2* (Transglutaminase 2), *IGLV1-51* (Immunoglobulin Lambda Variable 1–51), *LINC01010* (Long Intergenic Non-Protein Coding RNA 1010), and *CDK1* (cyclin-dependent kinase 1). Overall, our findings suggest that targeting MyD88 not only effectively downregulates known systemic inflammatory mediators but also could potentially inhibit upregulated genes in the RA synovium, preventing synovitis and joint degeneration.

## Discussion

Genome wide studies represent the most powerful and valuable tools to identify global transcriptional changes and dysregulated biological processes in RA. These approaches have allowed molecular stratification of RA patients and resulted in the identification of novel therapeutic targets (Teixeira et al., 2009; Shchetynsky et al., 2017; Lewis et al., 2019; Lee et al., 2020). However, an important limitation of these studies has been the sample variability in terms of differences in the administration of DMARDs. This limitation may have affected the gene expression signatures and interfered with the interpretation of the fundamental role of some genes in RA pathogenesis. Only a few studies have considered the analysis of treatment-naïve RA patients to identify gene expression signatures in response to treatment and to characterize better the RA transcriptional profiles obtaining insightful results (Shchetynsky et al., 2017; Boutet et al., 2021; Wang et al., 2021).

In this study, we performed RNA-seq analysis from DMARDs-naïve RA patients to decipher the effect of MyD88 inhibition independent on the impact of DMARDs. Due to the large number of genes overexpressed in our data set, the presentation of single genes was unsuitable. Instead, to get a functional overview and integration of the complex gene expression changes, we showed a global view of DE gene signatures and focused on pathways associated with those overexpressed genes. Despite the potential differences in the percentages of various cell types between the PBMC, the differentially expressed genes from healthy PBMC, RA PBMC and the PBMC under various treatments clustered together into their respective groups suggesting that our sample sets are representative of their treatment group or condition. As expected, the upregulated IPA-predicted disease associations in our study related to rheumatic disease, systemic autoimmune disorder, invasive tumor, psoriasis, and inflammation of joint were consistent pathogenic hallmarks in RA. Known canonical pathways in RA such as chemoattraction promoted by IL-8/CXCL8 signalling, granulocyte adhesion and diapedesis of immune cells, and high expression of degrading enzymes and growth factors in the osteoarthritis pathway were observed. We identified the neuroinflammation pathway as a primary mediator of pain in RA (Fuggle et al., 2014; Süß et al., 2020). In addition, the Aryl hydrocarbon receptor, known as Th17 cell differentiation promoter (Nakahama et al., 2011; Talbot et al., 2018), and TREM1 signalling involved in systemic and local inflammatory process activation (Kuai et al., 2009; Peng et al., 2019; Inanc et al., 2021), were also processes identified to be associated with the DMARDs-naïve RA PBMC. Based on our previous findings of the role of ST2825 in downregulating the release of proinflammatory cytokines in PBMC from healthy subjects (Ramírez-Pérez et al., 2020); we tested whether this chemical compound could modulate gene expression signatures and canonical pathways on PBMC from RA patients. Our findings indicate that ST2825 downregulates an enormous number of genes that are increased in RA patients compared with healthy subjects. Indeed, the genes downregulated by MyD88 inhibition were consistent with the IPA-predicted disease associations directly related to RA, such as systemic autoimmune syndrome, rheumatic disease, inflammation of joint, and rheumatoid arthritis. Both innate and adaptive immune responses were inhibited by ST2585.

The vital role of IL-17 in maintaining and promoting destructive processes in RA has been widely described (Tang et al., 2020; Miossec, 2021); however, only a few studies have explored the central role of MyD88 signalling in IL-17-driven inflammatory arthritis. In this regard, Abdollahi-Roodsaz et al. reported that IL-17 production and pathogenic changes such as joint swelling, inflammation and cartilage degeneration were significantly reduced in a MyD88 knockout mice model of inflammatory arthritis (Abdollahi-Roodsaz et al., 2012). Furthermore, MyD88 knockout also resulted in the downregulation of B cell signalling pathways and decreased production of autoantibodies. Other studies reported that the inhibition of MyD88 dimerization by ST2825 blocked the induction of plasma cell differentiation and antibody production from the PBMC of systemic lupus erythematosus (SLE) patients (Capolunghi et al., 2010). In corroboration with previous reports, we here showed that ST2825-mediated inhibition of MyD88 in PBMC also resulted in the downregulation of IL-17 signalling, antibody production, plasma cell activation and Th17 cell differentiation pathways. Indeed, our previous report suggested that ST2825 can decrease the secretion of IL-17A in PBMC from healthy donors; however, the results were not statistically significant (Ramírez-Pérez et al., 2020). Thus, we speculate that ST2825-mediated MyD88 inhibition could prevent the adverse effects of IL-17 signalling on RA PBMC; in parallel, we propose that future studies should be focused on identifying ST2825-mediated effects on Th17 cells from RA patients.

Critical canonical pathways typical for granulocytes, fibroblasts and endothelial cells in RA were downregulated on RA PBMC treated with ST2825. In support of this observation, a recent study identified a specific set of genes in blood samples from RA patients related to cartilage morphogenesis, endochondral bone growth and extracellular matrix organization. The authors concluded that those processes were observed due to the presence the presence of circulating preinflammatory mesenchymal (PRIME) cells in blood samples predicting flares in RA patients (Orange et al., 2020). It is also worth noting that PBMC are obtained by density centrifugation. On this matter, neutrophils are usually localized on the top of the erythrocyte pellet; however, in autoimmune diseases, a particular neutrophil population known as low-density granulocyte has been described (Carmona-Rivera and Kaplan, 2013; Wright et al., 2017; Ostendorf et al., 2019). This portion of low-density granulocyte is generally co-purified with the PBMC. We are not able to corroborate that those populations are present in our samples since we did not perform immune phenotyping; nevertheless, those previously reported findings may explain the identification of typical pathways for granulocyte adhesion and diapedesis, fibroblasts, or endothelial cells in PBMC samples.

MyD88 has also shown promising results by downregulating cytokines and chemokines and modulating LPS-induced mechanical hyperalgesia in the joint (Guerrero et al., 2016). Several large-scale transcriptomic changes have been identified in different joint-resident cell types under IL-1β stimulation (Gao et al., 2021; Haque et al., 2021). For this reason, inhibition of IL-1β and LPS signalling pathways is imperative. Our study identified overlapping of downregulated genes by the effect of ST2825 under distinct inflammatory conditions: 1) RA PBMC, LPS-stimulated RA PBMC, and 3) IL-1β-stimulated RA PBMC, where 461, 350 and 263 genes were significantly downregulated, respectively.

Our analysis revealed 40 commonly downregulated genes among conditions. We took advantage of an RNA-seq published data set from the Accelerating Medicines Partnership Rheumatoid Arthritis (RA) Phase I project (Zhang et al., 2019) to identify whether these genes may play a role in T and B cells, monocytes, and fibroblasts from RA synovium and shot listed 8 MyD88 target genes with potential roles in RA synovium. One of the shot listed genes *MMP9*, is known to be highly expressed in synovial fibroblasts in RA with therapeutic potential (Gossage et al., 2018; Shatunova et al., 2020). *CXCL9* was significantly overexpressed in fibroblasts and monocytes from the RA synovium and according to previous reports, its knockdown by exosomes containing miR-320a suppressed the activation, migration, and invasion of RA-FLS and decreased the arthritis index and inflammatory score in the CIA model (Meng and Qiu, 2020). We found that *MZB1* was highly upregulated in T cells and monocytes from leukocyte-rich RA synovium compared with OA. Although the role of *MZB1* in RA is yet to be clarified; its previously described role in SLE during the maintenance of splenic marginal zone B cells, plasma cells and autoantibody production (Miyagawa-Hayashino et al., 2018), suggest this molecule could play a central role in maintaining ectopic lymphoid structures, in the RA synovium. Fucosyltransferase 7 is (*FUT7*) is yet another MyD88 common target that was significantly increased in monocytes from RA synovial tissue. Zhang *et al.* showed that *FUT7* might play a role in inducing monocyte-endothelial adhesion, promoting atherosclerosis progression (Zhang et al., 2018), and FUT7 knockdown inhibited cell proliferation, migration, and invasion in metastatic follicular thyroid carcinoma cell lines (Qin et al., 2020). We, therefore, predict that *FUT7* may play a role in the proliferation, migration, and invasion of synovial fibroblasts and monocyte infiltration.

The other MyD88 target genes that were upregulated in RA synovium were *TGM2* (Transglutaminase 2), *CDK1* (Cyclin-dependent kinase 1), *IGLV1-51* (Immunoglobulin lambda variable 1–51) and *LINC01010* (long-non coding RNA *LINC01010*)*.* In support of a role for *TGM2* in RA, its knockdown resulted in a reduction of cartilage degradation and invadopodia formation in CIA model (Lauzier et al., 2012). Interestingly, high expression of *CDK1* positively correlated with interferon type 1 (IFN-1) serum levels and presence of anti-citrullinated protein antibodies (ACPA), which suggests a possible pathogenic role for *CDK1* in RA (Fattah et al., 2020). Finally, the roles of *IGLV1-51* and *LINC01010* in RA are yet to be defined.

Some limitations in our study were sample size sequencing; future studies must consider evaluation of a greater number of DMARDs-naïve RA patients in order to obtain a more robust estimation of ST2825 effects. The treatment of the patients in our data set with other non-DMARDs drugs could be an important variable as well. Our study did not take into consideration the disease stage or disease activity, which are relevant clinical variables in RA. Another limitation of the study is that the bulk RNA-sequencing of the unsorted PBMC population does not reveal the identity of the cell type that is most responsive to ST2825 treatment since immune phenotyping was not performed. The differential percentages of CD4^+^ T cells, CD8^+^ T cells, B cells, classical, intermediate, and non-classical monocytes, DCs, NK cells could potentially dictate the response to ST2825 in each individual patient. Therefore, future studies must consider the analysis of single-cell RNA sequencing to decipher whether specific immune cell populations contribute to the downregulation of inflammatory mediators and pathogenic processes observed in this study. Finally, although our analysis *in silico* revealed distinct genes that might be downregulated by ST2825 in the inflamed synovium of RA patients, this analysis might not represent the biological response of stromal and immune cells within the joint and further analysis should be carried out to corroborate these findings.

In summary, our study provides comprehensive evidence supporting the potential application of the MyD88 inhibitor, ST2825, as a modulator of systemic inflammatory processes in PBMC from DMARDs-naïve RA patients. Our indepth analysis of RNA-seq data will serve as a valuable resource containing the inflammatory gene expression signatures and pathogenic processes regulated by MyD88. Our findings also suggest that ST2825 might potentially downregulate crucial genes overexpressed in the RA synovium and function as an emerging therapeutic strategy for RA patients.

## Data Availability

The datasets presented in this study can be found in online repositories. The names of the repository/repositories and accession number(s) can be found below: Gene Expression Omnibus (GEO/NCBI); GSE189136.
