# Supplementary material for: Targeting MyD88 Downregulates Inflammatory Mediators and Pathogenic Processes in PBMC From DMARDs-Naïve Rheumatoid Arthritis Patients
Source: Front Pharmacol. 2021 Dec 23;12:800220. doi: 10.3389/fphar.2021.800220 (PMC8735861; doi:10.3389/fphar.2021.800220)
Supplement: Supplementary file 3 [file Table1.DOCX]

| **Supplementary table 1.** Quality control testing results from PBMC samples used in our study. | | | | | |
| --- | --- | --- | --- | --- | --- |
| **Sample** | **Age (years)** | **Gender** | **Viral testing *** | **DMARD** | **Other treatment** |
| **RA PBMC 2** | 42 | Female | Negative | NA | Ibuprofen, Megestrol |
| **RA PBMC 3** | 54 | Male | Negative | NA | Levothyroxine |
| **RA PBMC 4** | 37 | Female | Negative | NA | Rufinamide |
| **HS PBMC 1** | 42 | Male | Negative | NA | NA |
| **HS PBMC 4** | 71 | Female | Negative | NA | NA |
| RA, Rheumatoid arthritis patient. HS, Healthy subjects. PBMC, Peripheral blood mononuclear cells. DMARD, Disease-modifying antirheumatic drugs.  *HIV-1, HIV-2, HBV, HCV. | | | | | |
