# Supplementary material for: Targeting MyD88 Downregulates Inflammatory Mediators and Pathogenic Processes in PBMC From DMARDs-Naïve Rheumatoid Arthritis Patients
Source: Front Pharmacol. 2021 Dec 23;12:800220. doi: 10.3389/fphar.2021.800220 (PMC8735861; doi:10.3389/fphar.2021.800220)
Supplement: Supplementary file 4 [file Table2.DOCX]

| **Supplementary Table 2. List of 40 downregulated genes by ST2825 among conditions.** | | | |
| --- | --- | --- | --- |
| Gene |  |  |  |
| *AL139385.1*  *C017002.1*  *CD163*  *CDK1*  *CTSW*  *CXCL9*  *DNASE2B*  *FAM111B*  *FPR3*  *FUT7*  *IFNG-AS1*  *IGHG3*  *IGHV1-8*  *IGKV1-12*  *IGKV1D-16*  *IGKV2-24*  *IGLV1-51*  *IGLV2-14*  *IGLV8-61*  *LCNL1*  *LINC01010*  *MMP9*  *MRC1L1*  *MS4A4A*  *MSR1*  *MYBL2*  *MZB1*  *NMRK2*  *OCSTAMP*  *RP11-1136G11*  *RP11-134G8.6*  *RP11-173P15.5*  *RP11-327F22.2*  *RP11-63M22.2*  *RRM2*  *SERPINB2*  *SERPINE1*  *SPP1*  *SZT2-AS1*  *TGM2* | lncRNA  lncRNA  Scavenger receptor cysteine-rich type 1 protein M130  Cyclin-dependent kinase 1  Cathepsin W  C-X-C motif chemokine 9  Deoxyribonuclease-2-beta  Serine protease FAM111B  N-formyl peptide receptor 3  Alpha-(1,3)-fucosyltransferase 7  IFNG Antisense RNA 1  Immunoglobulin heavy constant gamma 3  Immunoglobulin heavy variable 1-8  Immunoglobulin kappa variable 1-12  Immunoglobulin kappa variable 1D-16  Immunoglobulin kappa variable 2-24  Immunoglobulin lambda variable 1-51  Immunoglobulin lambda variable 2-14  Immunoglobulin lambda variable 8-61  Lipocalin-like 1 protein  lncRNA  Matrix metalloproteinase-9  Macrophage mannose receptor 1  Membrane-spanning 4-domains subfamily A member 4A  Macrophage scavenger receptor types I and II  Myb-related protein B  Marginal zone B- and B1-cell-specific protein  Nicotinamide riboside kinase 2  Osteoclast stimulatory transmembrane protein  LincRNA  LincRNA  LincRNA  LincRNA  LincRNA  Ribonucleoside-diphosphate reductase subunit M2  Plasminogen activator inhibitor 2  Plasminogen activator inhibitor 1  Osteopontin  SZT2 Antisense RNA 1  Protein-glutamine gamma-glutamyltransferase 2 | | |
| lncRNA, Long noncoding RNA. LincRNA, Long intervening/intergenic noncoding RNA | | | |
