# Supplementary material for: Targeting MyD88 Downregulates Inflammatory Mediators and Pathogenic Processes in PBMC From DMARDs-Naïve Rheumatoid Arthritis Patients
Source: Front Pharmacol. 2021 Dec 23;12:800220. doi: 10.3389/fphar.2021.800220 (PMC8735861; doi:10.3389/fphar.2021.800220)
Supplement: Supplementary file 5 [file Table3.DOCX]

| **Supplementary table 3.** Description of PBMC bulk RNA-seq samples. | |
| --- | --- |
| **Condition** | **Sample sequenced** |
| **Healthy Untreated** | PBMC CS1 UTx  PBMC CS4 UTx |
| **RA Untreated** | PBMC RA3 UTx  PBMC RA4 UTx |
| **RA + ST2825** | PBMC RA2 ST2825  PBMC RA3 ST2825  PBMC RA4 ST2825 |
| **RA + LPS** | PBMC RA2 LPS  PBMC RA3 LPS  PBMC RA4 LPS |
| **RA + LPS + ST2825** | PBMC RA2 LPS ST2825  PBMC RA3 LPS ST2825  PBMC RA4 LPS ST2825 |
| **RA + IL-1β** | PBMC RA2 IL-1β  PBMC RA3 IL-1β  PBMC RA4 IL-1β |
| **RA + IL-1β + ST2825** | PBMC RA2 IL-1β ST2825  PBMC RA3 IL-1β ST2825  PBMC RA4 IL-1β ST2825 |
| RA, DMARDs-naïve rheumatoid arthritis patient. CS, Healthy subjects. PBMC, Peripheral blood mononuclear cells. DMARD, Disease-modifying antirheumatic drugs. LPS, lipopolysaccharide. IL-1β, interleukine-1β. ST2825, MyD88 dimerization inhibitor. UTx, untreated. | |
